# A Novel Cell Penetrating Peptide for the Differentiation of Human Neural Stem Cells

**DOI:** 10.3390/biom8030048

**Published:** 2018-07-09

**Authors:** Weili Ma, Geun-woo Jin, Paul M. Gehret, Neil C. Chada, Won Hyuk Suh

**Affiliations:** Department of Bioengineering, College of Engineering, Temple University, Philadelphia, PA 19122, USA; weili.ma@temple.edu (W.M.); geunwoo.jin@samyang.com (G.-w.J.); pgehret@temple.edu (P.M.G.); neil.chada@temple.edu (N.C.C.)

**Keywords:** cell penetrating peptide, neural stem cell, retinoic acid, differentiation, cytotoxicity, endocytosis

## Abstract

Retinoic acid (RA) is a bioactive lipid that has been shown to promote neural stem cell differentiation. However, the highly hydrophobic molecule needs to first solubilize and translocate across the cell membrane in order to exert a biological response. The cell entry of RA can be aided by cell penetrating peptides (CPPs), which are short amino acid sequences that are able to carry bioactive cargo past the cell membrane. In this work, a novel cell penetrating peptide was developed to deliver RA to human neural stem cells and, subsequently, promote neuronal differentiation. The novel CPP consists of a repeating sequence, whose number of repeats is proportional to the efficiency of cell penetration. Using fluorescence microscopy, the mode of translocation was determined to be related to an endocytic pathway. The levels of β-III tubulin (Tubb3) and microtubule associated protein 2 (MAP2) expression in neural stem cells treated with RA conjugated to the CPP were assessed by quantitative immunocytochemistry.

## 1. Introduction

Neurogenesis is an important biological process under intense investigation. Neurodegenerative diseases such as Alzheimer’s and Parkinson’s are devastating due to the fact that neurons cannot be replaced in the diseased areas of the brain [1]. The generation of new neurons from stem cells, however, has the potential to become a viable treatment option for patients suffering from neurodegenerative diseases [2,3]. Retinoic acid (RA) is an important bioactive lipid that has been shown to induce neurogenesis in stem cells, both in vitro and in vivo [4,5,6,7,8]. To exert its effects, RA must be internalized into cells, where it binds to the nuclear retinoic acid receptor (RAR) and retinoid X receptor (RXR). RAR heterodimerizes with RXR and binds to the retinoic acid response elements (RAREs), which activate transcription factors to induce neurogenesis [9,10]. Although RA is a potent morphogen, its efficacy is limited because of its low solubility (<0.2 µM) in aqueous environments [11]. There are several methods to overcome this drawback, such as encapsulation into nanoparticles [12,13,14] and microparticles [15,16], or conjugation to water-soluble polymers [17,18]. However, the concentration of RA being delivered will need to be monitored carefully, as high concentrations can induce developmental toxicity [19,20]. Because of the potential toxicity of RA, transient delivery methods can be used to control the dosage more effectively. Furthermore, RA has been shown to activate differentiation in stem cells either through long-term and low concentration treatments or single dose, high concentration treatments [21].

Cell penetrating peptides (CPPs) are short amino acid sequences that are able to translocate past the cell membrane [22]. The degradation of CPPs occurs rapidly because of it being a biological polymer, so any bioactive cargo delivered carries a transient effect [23,24]. The solubility of hydrophobic molecules can also be enhanced by conjugation to CPPs [25]. The earliest cell penetrating peptides were derived from natural proteins, such as the transactivator of transcription (TAT) peptide from human immunodeficiency virus 1 (HIV-1) and Penetratin (Antp) from antennapedia [26,27,28]. These CPPs interact with the negatively-charged cell membrane through arginine-rich, positively-charged domains [29]. More recently, several synthetic CPPs have been developed. These are generally amphipathic (or amphiphilic) and can interact with the cell membranes through either positive-charge, hydrophobicity, or both of these properties [30]. Amphipathic CPPs are advantageous in that more types of cargo can be delivered, such as negatively-charged cargos (i.e., nucleic acids), which would neutralize positively-charged CPPs and thwart their ability to penetrate cell membranes [31].

**Figure 1:**
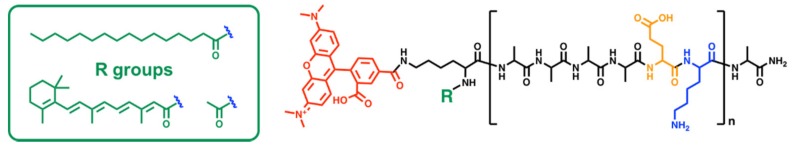
Chemical structure of PepB. Amine terminus R group (green): (I) acetyl (Ac), (II) palmitoyl (C16), or (III) retinoyl (RA); red: rhodamine dye; orange: carboxylic acid (negative charge); blue: primary amine (positive charge). The repeat sequence (AAAAEK) is repeated up to three times (*n* = 1, 2, 3) for this study.

In this study, a novel synthetic CPP called PepB was developed to facilitate the delivery of RA to human neural stem cells (hNSCs) and, subsequently, promote neuronal differentiation. Unlike other CPPs, PepB is a very short AAAAEK (six amino acid) sequence comprising four alanine (A, Ala), one glutamic acid (E, Glu), and one lysine (K, Lys) units. The PepB sequence can be repeated (Figure 1) to modulate the structure, size, solubility, toxicity, and cell penetration. The base PepB structure flanks (multiple) hydrophobic units with two oppositely charged hydrophilic residues, which resembles amyloid-like peptides, such as KLVFFAE [32,33,34], but is shorter in sequence and uses only three types of amino acids. We constructed nine different PepB structures (Figure 1 and Table 1) to study their biocompatibility and cellular incorporation (cell penetration) abilities. We, in addition, utilized RA-PepB_3_ to specifically test for its ability to differentiate hNSCs. We utilized human ReNcell VM (RVM, from ventral mesencephalon) cells for this latter study, which are from the midbrain (mesencephalon) area and have been recently utilized in stem cell engineering work [35,36,37,38].

## 2. Materials and Methods

*N*,*N*-Dimethylformamide (DMF, D119), Methylene Chloride (DCM, D37), Acetonitrile (ACN, A998), Methanol (MeOH, A452), Diethyl Ether (E134), Dimethyl Sulfoxide (DMSO), Sulfuric Acid (A300), Hydrogen Peroxide (H325), 4′,6-Diamidino-2-Phenylindole Dihydrochloride (DAPI, D1306), PrestoBlue^TM^ (A13262), and CellLight^®^ Lysosome-GFP (BacMam 2.0, C10596) were purchased from Thermo Fisher Scientific (Waltham, MA, USA). Cell Counting Kit-8 (CCK-8) was purchased from Dojindo Molecular Technologies (Rockville, MD, USA), Inc. Triisopropylsilane (TIPS, 233781), 1,8-Diazabicyclo[5.4.0]undec-7-ene (DBU, 139009), Palmitic Acid (C16, P5585), Phosphotungstic Acid Hydrate (PTA, P4006), and Donkey Serum (D9663) were purchased from Sigma Aldrich (St. Louis, MO, USA). Hydroxybenzotriazole (HOBt, CXZ010), 2-(1H-benzotriazol-1-yl)-1,1,3,3-tetramethyluronium Hexafluorophosphate (HBTU, CXZ020), Fmoc-Ala-OH (AFA101), Fmoc-Lys(Boc)-OH (AFK105), Fmoc-Lys(Dde)-OH (AFK140), Fmoc-Glu(OtBu)-OH (AFE105), and Trifluoroacetic Acid (TFA, CXZ035) were purchased from AAPPTec (Louisville, KY, USA). 4-Methylpiperidine (4-MePip, 137350) was purchased from BeanTown Chemical (Hudson, NH, USA). 5(6)-Carboxytetramethylrhodamine (TAMRA, Cat No. 361) and 5-Carboxy-X-rhodamine (ROX, Cat No. 381) were purchased from AAT Bioquest, Inc (Sunnyvale, CA, USA). Laminin (L2020), ReNcell VM (RVM, SCC008), ReNcell Maintenance Media (RMM, SCM005), Accutase (SCR005), Epidermal Growth Factor (EGF, GF144), Basic Fibroblast Growth Factor (bFGF, GF003), EmbryoMax^®^ Dulbecco’s Phosphate Buffered Saline (PBS, BSS-1006-B), Dulbecco’s Modified Eagle’s Medium with Ham’s F12 Nutrient Mixture (DMEM/F12, DF-041-B), β-III Tubulin antibody (AB9354 and MAB1637), MAP2 antibody (AB5622), FITC-Labeled Secondary Antibody (AP182F), and Cy5-Labeled secondary antibody (AP192C) were purchased from Millipore Sigma (Burlington, MA, USA). Penicillin-Streptomycin (Pen-Strep, 30-002-CI) was purchased from Corning (Corning, NY, USA). Paraformaldehyde (PFA, AC41678), *N*,*N*-Diisopropylethylamine (DIPEA, 115221000), Acetic Acid (Ac, 423220025), and All-Trans-Retinoic Acid (RA, 207340050) were purchased from Acros Organics (Part of Thermo Fisher Scientific). Cell culture related plasticware including T25; T75; well plates (BioLite, Falcon, or Nunc products); serological pipettes (Fisher Brand); pipettes tips (0.1–10 µL, 20–200 µL, 100–1250 µL sizes, Fisher Brand or Corning); and 1.5 mL, 15 mL, and 50 mL conical centrifuge tubes were purchased from Thermo Fisher Scientific or VWR (Radnor, PA, USA). Nunc Lab-Tek II 8-well Chambered Coverglass with non-removable wells (Cat. No. 155409) and Millipore EZ Slides (PEZGS0816) were purchased from Thermo Fisher Scientific. UV-Star Microplates (96 wells, half area, 675801) were purchased from Greiner Bio-One (Monroe, NC, USA). Disposable syringes (i.e., 1 mL, 5 mL), 0.22 µm filter flasks, and 0.22 µm syringe filters were purchased from Thermo Fisher Scientific or VWR. Transmission Electron Microscopy (TEM) grids were purchased from Ted Pella, Inc. (Redding, CA, USA).

### 2.1. Peptide Synthesis

All peptide sequences were synthesized by fluorenylmethyloxycarbonyl chloride (Fmoc) solid phase peptide synthesis (SPPS) methodology using 20 mL disposable plastic reaction vessels (Torviq, Niles, MI, USA) or 50 mL glass reaction vessels (ChemGlass, Vineland, NJ, USA) incorporating porous frits. An incubating shaker fitted with a 50 mL tube rack (Fisher Scientific) or a wrist shaker (Burrell Scientific, Pittsburgh, PA, USA) was utilized for applying agitation to the reaction vessels. The SPPS reactions were carried out at room temperature at 400 RPM, unless otherwise stated. The major steps include (1) swelling, (2) deprotection, (3) coupling, and (4) cleaving [39]. Rink amide resin was swollen in DCM for 30 min. The resin was washed with DMF three times for 5 min each. Fmoc was deprotected using a solution of 2% (*v*/*v*) DBU and 2% (*v*/*v*) 4-MePip in DMF. The deprotection solution was refreshed every 20 min for a total of 1 h. The deprotected resins were washed with DMF three times for 5 min each. A few resins were extracted using a micropipette for Kaiser testing. The Kaiser test was performed by incubating the resins at 110 °C with a drop each of solution A (50 mg/mL ninhydrin in ethanol), solution B (0.2 mM potassium cyanide in pyridine), and solution C (0.8 g/mL phenol in ethanol). If the Kaiser test was positive for free amines, the resins went on to conjugation. If the Kaiser test came back negative, another cycle of deprotection was performed. For conjugation, six molar equivalents of DIPEA and 3 molar equivalents of HOBt, HBTU, and the next Fmoc-conjugated amino acid or chemical in the sequence were dissolved in 10 mL of DMF and then mixed into the reaction vessel. The conjugation reaction was carried out for at least 4 h. Afterwards, resins were washed with DMF three times for 5 min each. Another Kaiser test was conducted to check for conjugation. The deprotection and conjugation reactions were repeated until the peptide sequence was completed. A final deprotection of the 1-(4,4-dimethyl-2,6-dioxocyclohex-1-ylidene)-3-ethyl (Dde) groups was performed for 1 h using a solution of 2% (*v*/*v*) hydrazine in DMF. After confirming deprotection by Kaiser testing, dye was conjugated. The peptides were cleaved using a solution of 2.5% (*v*/*v*) TIPS and 2.5% (*v*/*v*) water in TFA with shaking at 400 RPM for 1 h.

### 2.2. Peptide Purification and Isolation

The acid-cleaved peptides were air-dried overnight. The peptides were washed with ice-cold diethyl ether by ultrasonicating at 4 °C for 30 min. The peptides were then centrifuged at 7000× *g* at 4 °C for 15 min to pelletize. The supernatant was discarded, and the ether wash was repeated two more times. The remaining ether was air-dried overnight. The peptides were resuspended in a solution of 0.1% (*v*/*v*) TFA in water and sonicated until completely solubilized. Palmitic acid tail peptides were solubilized in 0.1% (*v*/*v*) TFA in methanol. The peptide solution was filtered through a 0.22 µm membrane and separated using reverse phase high performance liquid chromatography (HPLC; Waters 2545 quaternary gradient module, Waters 2996 photodiode array detector, Waters fraction collector 3, and Luna Phenomenex C8 column) using 0.1% (*v*/*v*) TFA in water and 0.1% (*v*/*v*) TFA in acetonitrile as the mobile phases. Acetonitrile was substituted with methanol for peptides with palmitic acid tails. The collected peptide solutions were lyophilized and stored desiccated at −20 °C until use.

### 2.3. Mass Spetrometry

Successful synthesis of peptides was confirmed by molecular weight analysis utilizing electrospray ionization time-of-flight mass spectrometry (ESI-ToF MS, Agilent 6520, Santa Clara, CA, USA). Lyophilized peptides were solubilized in a solution of 50/50 water/acetonitrile, containing 0.1% (*v*/*v*) formic acid to a final concentration of 100 µM. The peptides were filtered through a 0.22 µm membrane and 1 nL was injected using a 175 V fragmentor voltage. The mass spectra were obtained in a positive ion mode for exact mass analysis after background subtraction. For matrix-assisted laser deposition/ionization time-of-flight mass spectrometry (MALDI-ToF MS, Bruker Autoflex, Billerica, MA, USA), the peptide samples were prepared at 0.5 mg/mL in 50:50 methanol:water mixture containing 0.1% TFA. The samples were embedded in an α-Cyano-4-hydroxycinnamic acid (CHCA) matrix.

### 2.4. Circular Dichroism

Circular dichroism (CD) spectra were obtained for the PepB_3_ variants. PepB_3_ solutions between 80 to 140 µM were prepared by dissolving the lyophilized powders in distilled water. The CD spectra were acquired on an AVIV 410 CD Instrument. Each sample was analyzed between 195 to 240 nm using a bandwidth of 1 nm, scan time of 10 s/nm, and a total of three scans. The final spectra were averaged, smoothed, and plotted using MATLAB (MathWorks, Natick, MA, USA).

### 2.5. Transmission Electron Microscopy

PepB_3_ variants were prepared for transmission electron microscopy (TEM) using a negative staining protocol. Lyophilized peptides were dissolved in water at varying concentrations and 2 µL was applied to a TEM grid. After air drying for 30 min, 5 µL of 0.1% *w*/*v* phosphotungstic acid (pH 7.4, 0.2 µm filtered) was applied to the TEM grids for 10 s. The solution was blotted with filter paper and the grids were air dried another 30 min before being placed under vacuum for 12 h. Images were taken using a JEOL JEM 1400 TEM microscope (JEOL USA, Peabody, MA, USA) fitted with a Gatan UltraScan 1000 CCD camera (Gatan, Pleasanton, CA, USA).

### 2.6. Cell Culture and Differentiation

ReNcell VM immortalized human neural stem cells were cultured for the biological experiments under standard incubation conditions (37 °C, 5% CO_2_). Laminin coated tissue culture flasks were prepared by diluting laminin to 20 µg/mL in DMEM/F12 media and incubating for at least 4 h. Prior to cell seeding, the laminin solution was removed, and fresh proliferation media was added. The RVM cells were maintained in ReNcell maintenance media (Millipore), supplemented with 1% (*v*/*v*) pen-strep, 20 ng/mL EGF, and 20 ng/mL bFGF. The media were refreshed every 24–48 h for RVM during proliferation. The cells were passaged when 70% confluency was reached by incubating with Accutase at room temperature for 5 min. The cells were pelletized by centrifugation at 200 × *g* for 5 min.

For the differentiation, 8-well chamber slides were coated with 20 µg/mL laminin in DMEM/F12 media. The laminin solution was removed prior to cell seeding. 2 × 10^4^ ReNcell VM between passages 8 and 10 were seeded in the 8-well chamber slides with 400 µL of proliferation media. The cells were grown to 70% confluency. Differentiation for RVM is initiated by growth factor withdrawal or in combination with RA or RA-PepB_3_. The media were replaced every 48–72 h for the duration of the differentiation. Compound treatment was only performed for one week, at which point all cells were commonly grown in growth factor withdrawal media.

### 2.7. Immunocytochemistry

Cells were fixed with 4% PFA at room temperature for 15 min. After rinsing with PBS, blocking and permeabilization were done simultaneously using 5% donkey serum and 0.3% Triton-X 100 in PBS for 30 min at room temperature. The cells were rinsed with PBS and primary antibodies were applied for 2 h at room temperature. The cells were rinsed again with PBS and secondary antibodies were applied for 1 h at room temperature. The nuclei were stained with DAPI (300 ng/mL in PBS) and the cells were imaged on an Olympus IX-83 microscope (Olympus USA, Center Valley, PA, USA), equipped with a Hamamatsu Orca-R2 camera (Hamamatsu Photonics, Hamamatsu City, Shizuoka, Japan). The cells positive for β-III tubulin and MAP2 were counted using ImageJ. The microscopy images were opened using the Olympus plug-in for ImageJ. The fluorescence channels were separated, background removed, and contrast enhanced. The number of DAPI positive cells were counted using an in-house coded nuclei counter. This macro was applied to all images for consistency. The channels were then re-merged and the cells positive for β-III tubulin and MAP2 were manually counted. The cells were considered positive only if the signal also overlapped with a DAPI signal.

### 2.8. Peptide Uptake and Flow Cytometry

To observe the uptake mechanism, 8-well chamber slides were coated with 20 µg/mL laminin in DMEM/F12 media. The laminin solution was removed prior to cell seeding. There were 2 × 10^4^ ReNcell VM between passages 6 and 10 that were seeded in each well, with 400 µL proliferation media. After 24 h, lysosomes were labeled by adding 2 µL of CellLight^®^ Lysosome-GFP, BacMam 2.0 into to the cell culture media followed by overnight incubation. The peptide stocks were prepared in sterile dimethyl sulfoxide (DMSO). The fluorescence microscopy images were taken at various time points after treatment with 1 µM RA-PepB_3_. For flow cytometry, 96-well plates were laminin coated and 2 × 10^4^ cells were seeded in each well, with 100 µL proliferation media (per well). The cells were incubated for 2 h to allow attachment. At the time of treatment, the peptide stock solutions prepared in ethanol were diluted to 1 µM in the cell culture media (final ethanol concentration <0.5%). The cells were incubated with peptides for 24 h. Afterwards, the cells were washed with PBS and the media was changed for live cell imaging. The peptide uptake was observed on an Olympus IX-83 microscope equipped with a Hamamatsu Orca-R2 camera, Prior automated stage (Prior Scientific, Rockland, MA), and a live cell instrument (LCI; Seoul, South Korea) Chamlide live cell imaging stage-top incubator system. The differential interference contrast (DICT) and fluorescence images were captured at 20 × magnification. After the live-cell imaging, the media were removed and 50 µL Trypsin EDTA was added to each well. The cells were detached by incubation for 5 min. After detachment, 100 µL of 4% PFA was added to each well to fix the cells for flow cytometry. The quantity of 5(6)-TAMRA labeled cells was measured on an Accuri C6 flow cytometer (Accuri Cytometers, Inc., Ann Arbor, MI, USA).

### 2.9. Quantification of Peptide Stock Solutions

Sterile peptide solutions for cytotoxicity testing were prepared from stock by diluting with deionized (DI) water and filtering through a 0.22 µm polytetrafluoroethylene (PTFE) membrane. Because of aggregations and decreased solubility in water at high concentrations, a standard curve was used to determine the concentrations of the filtered peptides. Briefly, 5 µL of sample or serial diluted peptide stocks with known concentrations prepared from weighed powder, were aliquoted into wells of a 96-well half area plate. Then, 45 µL of methanol was added to each well to fully solubilize all the peptides. Absorbance measurements at 554 nm (650 nm as reference) were taken on a Tecan infinite M200 Pro plate reader (Tecan Group, Männedorf, Switzerland). After the calculation of stock concentrations, peptides were further diluted with cell culture media for treatment.

### 2.10. Cytotoxicity

96-well plates were coated with 20 µg/mL laminin in DMEM/F12 media for at least 4 h in the incubator. The laminin solution was removed prior to the cell seeding. There were 10,000 ReNcell VM cells (passages below 15) that were seeded in each well, with 100 µL of RVM cell culture media supplemented with the growth factors bFGF and EGF. The attached cells were utilized between 2–18 h of stabilization.

For the PepB series (Figure 1 and Table 1) testing, the RVM cells were incubated with peptides for 24 h. After 24 h, the cells were washed twice with stem cell culture media and treated with CCK-8 (WST-8) reagent (or PrestoBlue^TM^). After incubation for 1 h, for WST-8, a Tecan infinite M200 Pro plate reader was utilized to acquire absorbance measurements at 450 nm (650 nm as reference) and normalized to the untreated controls.

For the retinoic acid long-term cytotoxicity study, an identical protocol was utilized. Briefly, a stock solution of RA was dissolved in DMSO at 2 mM, which was then sterile filtered through a 0.22 µm membrane. The final concentration was confirmed with a standard curve, by measuring the absorbances of known concentrations of RA at 351 nm (in methanol). The dilutions were performed in stem cell proliferation media and the cytotoxic effects were measured with PrestoBlue^TM^ every 48 h. The RA-containing media were refreshed after every reading. A PrestoBlue^TM^ solution was prepared by mixing it with the proliferation media at a 1:10 ratio. The cells were incubated with PrestoBlue^TM^ for 30 min, and fluorescence (560/590 nm excitation/emission wavelengths) was measured using a Tecan infinite M200 Pro plate reader. The intensities were normalized to untreated controls from day one.

### 2.11. Statistical Analysis

The statistical significance for treatment groups were determined using JMP Pro 13 (SAS Institute, Cary, NC, USA) and Microsoft Excel (Microsoft Corporation, Redmond, WA, USA). The Student’s *t*-test was performed and the resulting *p*-value less than 0.05 were considered significant. Biological experiments were performed at least three times independently.

## 3. Results

### 3.1. Peptide Characterization

All PepB variants (Figure 1) contained a conjugated rhodamine dye (e.g., TAMRA or ROX) and were synthesized using Fmoc SPPS [22]. The exact mass analysis performed using electrospray ionization time-of-flight mass spectrometry (ESI-ToF MS) showed matching *m*/*z* for all peptides (Table 2). The exact mass was also matched using matrix-assisted laser deposition/ionization time-of-flight mass spectrometry (MALDI-ToF MS) (Table 2).

**Figure 2:**
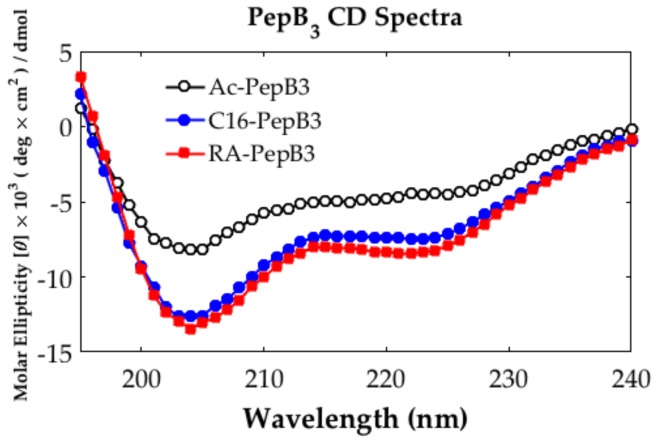
Circular dichroism (CD) spectra of PepB_3_ variants. All PepB_3_ variants exhibit a mixture of random coil and α-helical secondary structures. The lipidated PepB_3_ had more pronounced peak features compared to acetylated PepB_3_.

**Figure 3:**
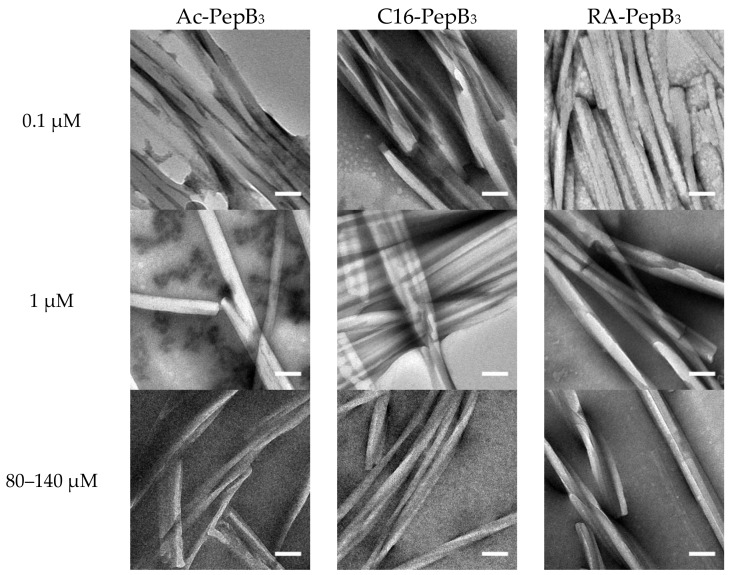
Transmission electron microscopy (TEM) images of PepB_3_ variants. (Column 1) Ac-PepB_3_, (Column 2) C16-PepB_3_, and (Column 3) RA-PepB_3_ all formed elongated micelles and 0.1 µM (1st row,), 1 µM (second row), and 80–140 µM (third row) concentrations, respectively. Scale bar = 100 nm; the peptide concentrations are divided into three categories, as denoted.

The PepB_3_ variants were probed for their secondary structures using CD. The peptides were dispersed in water at approximately 100 µM concentrations, which are recommended values for measuring peptides and peptide amphiphile samples for secondary structure analysis [40,41]. After the data processing and plotting in MATLAB, all PepB_3_ variants seem to contain a mixture of random coil and α-helical structures (Figure 2). We observed identical random coil and α-helical structures when analyzing the samples below 10 µM (data not shown). We calculated the % α-helicity values utilizing the % helix content equation provided in the literature [42,43], and the results show that RA-PepB_3_ is approximately 28% α-helix, while C16-PepB_3_ is approximately 25%, and Ac-PepB_3_ is approximately 16%. This result is consistent with the reported instances where lipidation increases helical secondary structural propensities in short peptides via micelle structure formation (high-order structure; Figure 3) [40,44].

The TEM analysis was performed at varying concentrations to see higher-order structures (Figure 3). The PepB_3_ variants were TEM analyzed after negative staining with 0.1% phosphotungstic acid. Ac-PepB_3_, C16-PepB_3_, and RA-PepB_3_ all formed elongated micelles with diameters ranging between 20 to 50 nm. We observed that higher-order micellar structures formed between 0.1 µM and 140 µM.

### 3.2. Peptide Concentration Quantification

**Figure 4:**
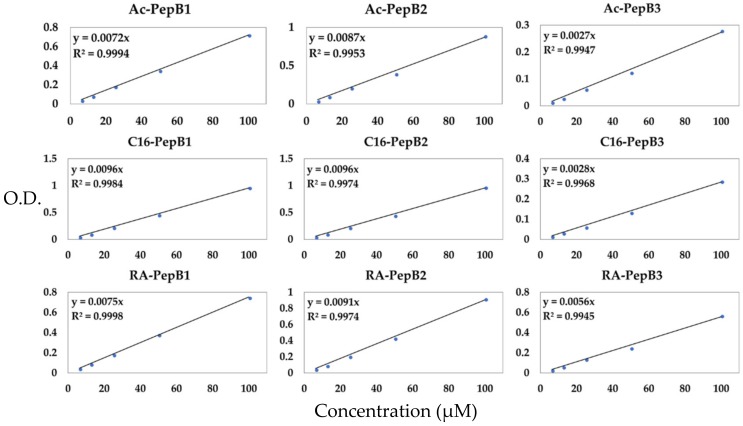
Standard curves to determine filtered peptide concentrations in cytotoxicity and differentiation experiments. *N* = 9 per standard. O.D.: optical density.

Due to potential aggregations of peptides in water prior to filter sterilization, the concentrations of peptides were re-measured using a standard curve generated using unfiltered peptides with known concentrations (Figure 4).

### 3.3. Cytotoxicity

**Figure 5:**
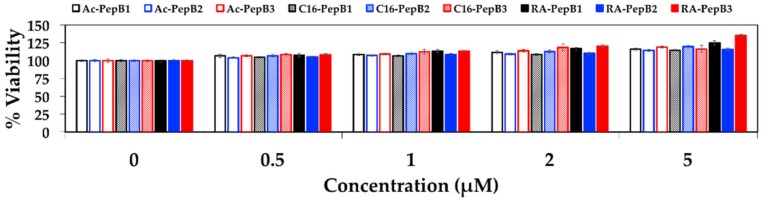
ReNcell VM (RVM, from ventral mesencephalon) cell viability measured with WST-8 after 24 h of treatment with varying concentrations and repeats of Ac-PepB, C16-PepB, and RA-PepB. *N* = 12; error bars depicted as standard error of the mean (SEM).

Nine PepB variants were determined to be non-cytotoxic between 0–5 µM concentrations for RVM cells (Figure 5). No significant decrease in the cell viability of RVM cells was observed after 24 h of treatment (incubation) in the stem cell media when tested with a water-soluble tetrazolium salt system [45].

**Figure 6:**
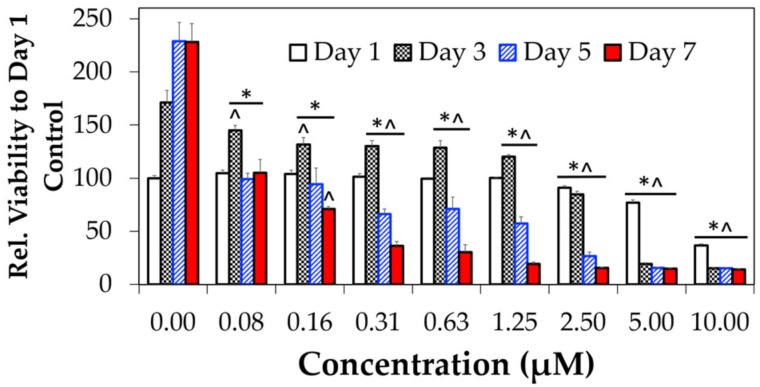
RA ReNcell VM (RVM) cell viability assessment results. Cell viability and proliferation profile measured with PrestoBlue^TM^ after treatment with varying concentrations of retinoic acid. *N* = 3; error bars depicted as standard deviation (SD); * *p* < 0.05 compared to same day (group) control (0 µM); ^ *p* < 0.05 compared to day one control (0 µM).

**Figure 7:**
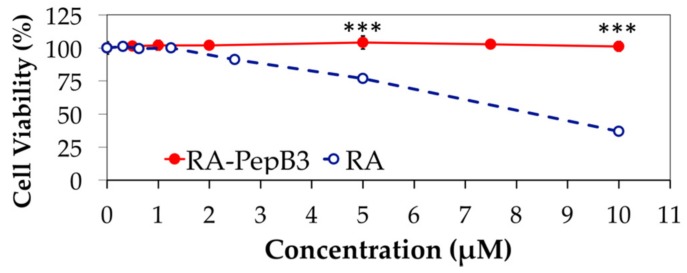
RVM Cell viability comparison between RA and RA-PepB_3_. Cell viability after treatment with varying concentrations of RA and RA-PepB_3_ were assessed utilizing either PrestoBlue^TM^ or Cell Counting Kit-8. *N* = 3; error bars depicted as SD; *** *p* < 0.001.

Retinoic acid showed considerable cytotoxicity (Figure 6 and Figure 7) that matches the reported observations [46,47], unlike our PepB variants. First, a long-term effects study (Figure 6) was conducted while the cells were subjected to differentiating conditions in the presence of RA. After 24 h of treatment (white bars), the cells treated with 5 and 10 µM RA showed significantly less proliferation than the controls. By day three (dark grey bars), all tested concentrations showed significantly reduced rates of proliferation. By day five (blue bars) and seven (red bars), even the cells treated with the lowest concentrations of RA (0.3125 and 0.625 µM) had significantly reduced levels of proliferation. These toxic concentrations somewhat match a recent microarray chip study reporting RA viability IC_50_ (half maximal inhibitory concentration) of 0.74 µM for undifferentiated RVM cells cultured in Matrigel [46]. Based on these findings, utilizing RA at 0.15 µM or above concentrations will be detrimental for conducting long-term studies. This is one of the reasons why we conducted long-term differentiation studies (see Section 3.5) at 0.1 µM RA condition and not anything higher.

Next, a short-term 24 h toxicity study was performed for RA between 0–10 µM concentrations (Figure 7) to compare with PepB_3_ modified RA. In this instance, the RVM cells were treated with RA for 24 h and then the toxicity was measured. For RA, the RVM cells started significantly dying at 5 µM, and, by 10 µM, more than 50% were killed off, while the RA-PepB_3_ treated RVM cells were fine. The reported IC_50_ value range for retinoic acid with RVM cells (cultured in three-dimensional [3D] alginate hydrogels) is approximately 10–15 µM [46,47], which was measured utilizing calcein vs. ethidium homodimer-1 staining, and our results somewhat match up. The conjugation of PepB_3_ peptide to RA attenuated toxic effects (reduced cytotoxicity) normally observed for RA at the higher concentrations past 1 µM (Figure 7).

### 3.4. Cell Uptake and Flow Cytometry

**Figure 8:**
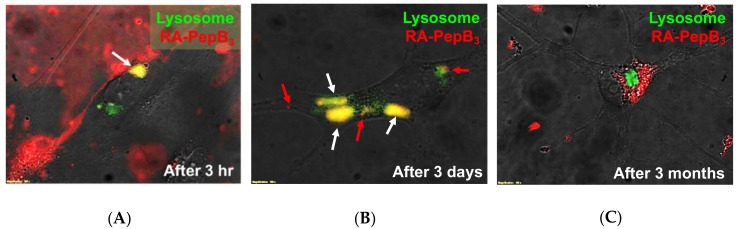
Live cell microscopy results for RA-PepB_3_ treated RVM Cells. RA-PepB_3_ was found localized to the lysosomes after (**A**) 3 h of treatment at 1 µM (white arrow, yellow region); (**B**) 3 days after initial treatment, RA-PepB_3_ was mostly localized to the lysosomes (white arrows, yellow region) but some have escaped into the cytosol (red arrows); and (**C**) after 3 months, the red-dye-labeled molecules have escaped the lysosome.

After treating the cells with 1 µM RA-PepB_3_, the lysosomes were labeled with CellLight^®^ Lysosome-GFP. RA-PepB_3_ was found in lysosomal vesicles after 3 h, indicated by the yellow color resulting from the overlap of red (rhodamine-dye) and green (GFP) (Figure 8A). After three days, most of the RA-PepB_3_ was found localized in the lysosomes, but some had escaped into the cytosol (Figure 8B). After three months, the red-dye-labeled molecules are no longer co-localized to the lysosomal compartments (Figure 8C).

**Figure 9:**
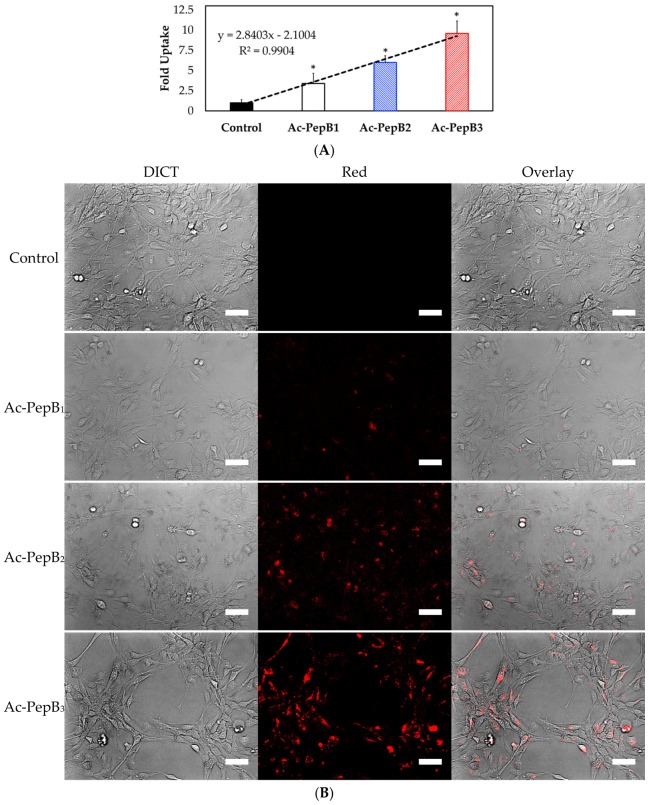
PepB series uptake results on RVM cells. (**A**) Flow cytometry results showed Ac-PepB repeat sequence-dependent cell uptake in RVM. (**B**) Live-cell fluorescence microscopy images of RVM after 24 h treatment with 1 µM Ac-PepB. *N* = 3; error bars depicted as SD; * *p* < 0.001; scale bar = 50 µm.

**Figure 10:**
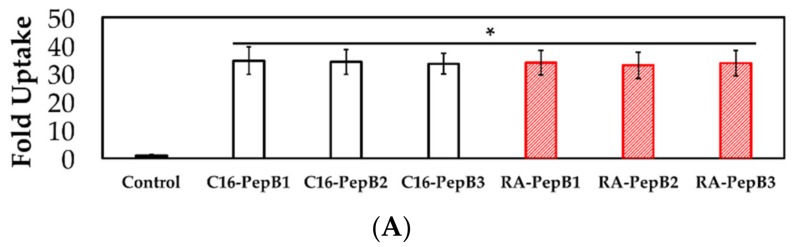

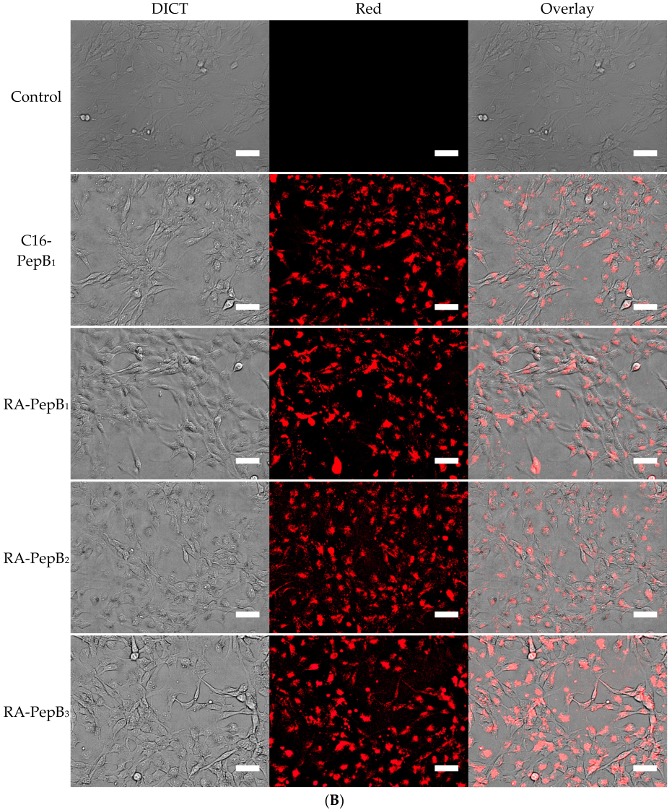
Lipidated PepB variant uptake results. (**A**) Lipidation of PepB significantly increased cell uptake in RVM, irrespective of the number of repeat-sequences. (**B**) Live-cell fluorescence microscopy images of RVM after 24 h treatment with 1 µM lipidated PepB. C16-PepB_2_ and C16-PepB_3_ uptake profiles are identical to C16-PepB_1_. *N* = 3; error bars depicted as SD; * *p* < 0.001; scale bar = 50 µm.

After 24 h of treatment at 1 µM, the flow cytometry results also indicated successful cell incorporation. Ac-PepB showed a PepB repeat-dependent increase in cellular uptake. When normalized to untreated cells, Ac-PepB had 3.4-, 6-, and 9.6-fold increased red fluorescence signal for PepB 1-, 2-, and 3-repeat variants, respectively (Figure 9). C16-PepB and RA-PepB showed a 34-fold increased uptake (82% of cells) compared with the control, regardless of the PepB repeat numbers (Figure 10).

### 3.5. Long-Term and Short-Term Differentiation Results

We tested RA-PepB_3_ for RVM differentiation over different time and concentration points. First, we conducted long-term experiments at 0.1 µM (low) concentrations, as RA kills RVM cells during differentiation at higher concentrations (Figure 6). In addition, this allowed us to hypothesis test whether the extra PepB_3_ sequence (extra 20-mer peptide) alters RA’s ability to differentiate hNSCs into neuronal cells.

**Figure 11:**
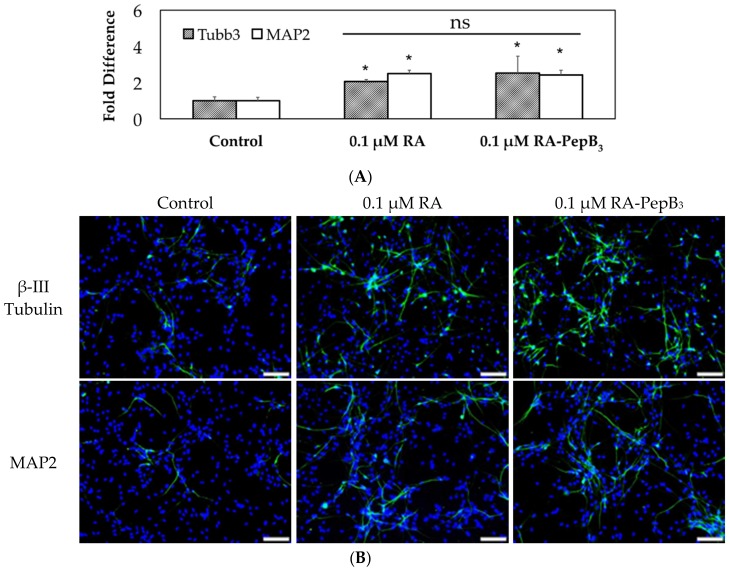
Long-term low-concentration RVM differentiation results. (**A**) Comparison of β-III tubulin and MAP2 expression levels in four-week differentiated RVM. ns: no significant. (**B**) Fluorescence microscopy images of ReNcell VM after four weeks of differentiation. Cells were stained for nuclei (DAPI; blue), β-III tubulin (green, top row), and MAP2 (green, bottom row). Control group cells were differentiated with only growth factor withdrawal (no additional compounds added). *N* = 3; error bars depicted as SD; * *p* < 0.05 compared to control expression levels; scale bar = 100 µm.

The RVM cells were differentiated by growth factor withdrawal or in combination with RA or RA-PepB_3_. The inducing compound treatments were performed during the initial one week, at which point all of the cells were commonly switched to growth factor withdrawn media. The RVM growth media (with no bFGF and no EGF) were replenished (replaced) every 48–72 h for the duration of the differentiation process. The four-week differentiation was stopped via fixation, utilizing 4% paraformaldehyde. These fixed RVM cells were stained for β-III tubulin and MAP2 after four weeks of differentiation. Compared to the induction of differentiation by growth factor withdrawal (control), β-III tubulin expression in RVM treated with 0.1 µM RA or 0.1 µM RA-PepB_3_ was 2- and 2.5-fold higher, respectively. (Figure 11A,B). The MAP2 expression was 2.5-fold higher than control for both 0.1 µM RA and 0.1 µM RA-PepB_3_ treated cells (Figure 11A,B). There were, however, no significant (Figure 11A) difference between RA vs. RA-PepB_3_ treatment conditions. We, therefore, can accept that the PepB_3_ addition to RA does not affect RA’s ability to differentiate hNSCs.

**Figure 12:**
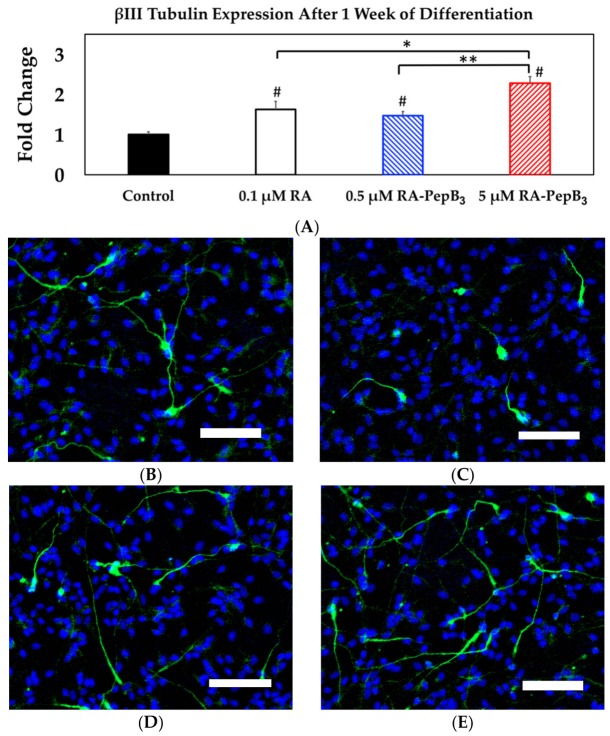
Short-term high-concentration RVM differentiation results. (**A**) Comparison of β-III tubulin levels in one week differentiated RVM. Fluorescence microscopy images of ReNcell VM after one week of differentiation were taken for (**B**) control, (**C**) 0.1 µM RA, (**D**) 0.5 µM RA-PepB_3_, and (**E**) 5 µM RA-PepB_3_. The cells were stained for nuclei (DAPI; blue) and β-III tubulin (green). Control group cells were differentiated by growth factor withdrawal only (no additional compounds). *N* = 3; error bars depicted as SEM; Student's *t*-test, * *p* < 0.05, ** *p* < 0.01, # *p* < 0.001 compared with control; scale bar = 100 µm.

Secondly, we tested whether the accelerated differentiation can be achieved utilizing higher concentrations of RA-PepB_3_. We altered the initial study’s four-week differentiation time point to one week and increased the RA-PepB_3_ concentrations. In this study, we technically had two control conditions. One was no treatment control and the other was 0.1 µM RA treatment condition. We tested whether 0.5 µM and 5 µM significantly increased the β-III tubulin expression after (only) one week of differentiation. The RVM cells were fixed at the one-week time point and stained for β-III tubulin (Figure 12). There was no statistical difference between 0.1 µM RA and 0.5 µM RA-PepB_3_ conditions (both showed 1.5-fold increased β-III tubulin expression compared to the no growth factor condition control). Increasing the dosage to 5 µM RA-PepB_3_, however, increased the β-III tubulin expression to 2.2-fold, which was statistically significant as shown (*p* < 0.05). All the treatment conditions showed a statistically significant increase in the β-III tubulin expression compared with the untreated controls.

## 4. Discussion

**Figure 13:**
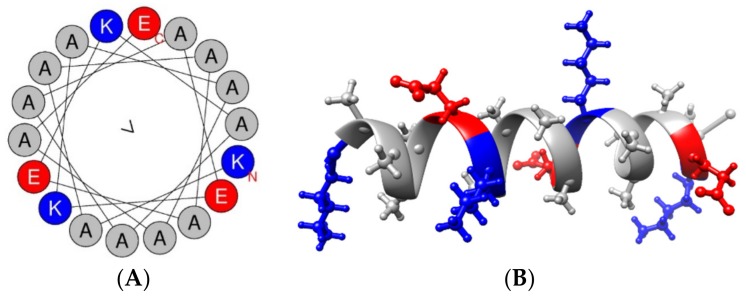
(**A**) Helical wheel of PepB_3_ generated using Heliquest. The base peptide sequence has a zero-net charge and hydrophobic moment. (**B**) Folding of Ac-PepB_3_ into an α-helix was predicted using PEP-FOLD3. For visualization, UCSF Chimera software [61] was utilized.

The most interesting and unique aspect of PepB is its repeating sequence (AAAAEK). Unlike some other synthetic CPPs [48,49,50], PepB does not carry a net positive charge because of charge balancing by the glutamic acid and lysine residues. Furthermore, PepB_3_ has a net hydrophobic moment of 0 µH according to Heliquest modeling (Figure 13A) [51]. Larger hydrophobic moments are thought to be associated with enhanced uptake efficiency [52], but since PepB has a zero net hydrophobic moment, the penetration could be related to its secondary structure [53]. CPPs that are able to form α-helices have been shown to have stronger membrane interactions compared to those forming β-sheets [54]. An α-helix is a right-handed screw-like structure that is stabilized by internal hydrogen bonding between the 1st and 4th amino acid residue (i, i + 4) [55]. A stable α-helical structure requires at least two to three turns of the helix, and thus 7 to 11 amino acid residues are required for stability [56]. Including the preceding alanine, each PepB repeat is six amino acids long (AAAAEK). The formation of a stable α-helical structure was predicted using the PEP-FOLD3 software for three repeats (Figure 13B) [57]. The Ac-PepB_3_ structure, however, is not 100% α helical based on the CD measurements (Figure 2)—it is approximately 16% α-helix and 84% random coil. The addition of lipids (palmitoyl, retinoyl groups) increases the α-helicity by approximately 9–12%, and these phenomena match reported examples [40,44]. Furthermore, alanine is known to be able to form stable α-helices [58,59,60].

The amount of uptake by ReNcell VM human neural stem cells was dependent on the number of PepB repeat sequences (Figure 9). This could be related to the increased stability of the α-helical structure due to the increasing numbers of hydrogen bonds. According to the CD spectra obtained for the PepB_3_ series (Figure 2), PepB exhibited a mixture of random coil and α-helix secondary structures in water, indicated by minima at 208 and 222 nm [62]. Adding lipid tails further increased the secondary structure, in accordance with the previously reported results on peptide amphiphiles [40,44]. The molar ellipticity peaks were more pronounced in the lipidated PepB samples compared with the acetyl-capped PepB. TEM images (Figure 3) show that the lipidated PepB was able to form high-aspect-ratio (elongated) micelles, indicative of self-assembling structures [63,64,65]. It is possible that these peptides are able to form a stable gel-like material at higher concentrations [66]. Protein and peptide lipidation is a well-established mechanism through which membrane penetration and cell signaling can be enhanced [67,68,69]. This effect is clearly seen by the lipidation of PepB with either palmitic acid or retinoic acid (Figure 10). The PepB variants tested in this study were found to be non-cytotoxic to RVM cells, even up to 10 µM (Figure 5 and Figure 7). Although this study only examined up to three repeats of Ac-PepB, more uptake may be possible by increasing the number of repeats. A linear regression was applied to the Ac-PepB uptake with high *R*^2^ (Figure 9A). Using the line equation, it was calculated that 13 repeat units would be necessary to match the level of uptake of lipidated PepB (34-fold). Thus, increasing the number of repeats may be a viable alternative to lipidation. Peptide synthesis, however, has several limitations that would make this difficult. The yield decreases with every conjugation, and the accepted limit is around 50 amino acids in length [70]. Nine repeats of PepB would go past this limit (54 mer). Furthermore, as the length increases, there are potential side reactions and aggregations, which can result in an even lower yield [71]. Finally, each conjugation requires the use of reagents in molar excess, which can rapidly increase the budget for longer peptide sequences. One alternative is to use recombinant synthesis, which would allow for larger peptides to be synthesized with the help of microorganisms. However, care must be taken as unexpected modification of the peptide can occur [72].

Retinoic acid is a potent morphogen used to differentiate stem cells towards the neural lineage [73,74]. First, it should be noted that RA is often used at concentrations beyond its reported solubility limit of <0.2 µM [11]. Aside from solubility issues, higher concentrations of RA have been shown to affect cell viability, albeit to varying degrees, depending on cell type [75,76,77,78,79,80,81]. The cytotoxicity results from this study suggest that the viability of ReNcell VM human neural stem cells is affected by RA at concentrations past 5 µM when treated for 24 h (Figure 7). Long-term treatment for seven days resulted in cytotoxicity, even at the lower concentrations of 0.15 µM (Figure 6). The growth inhibitory effect of RA is well-known in the field of cancer research [82,83,84]. RA-induced apoptosis is thought to be a result of mitochondrial dysfunction [85,86]. Interestingly, the conjugation of RA to PepB_3_ attenuated the cytotoxicity at higher concentrations (Figure 7). The RA cytotoxicity has previously been modulated by PEGylation [87]. To our knowledge, this is the first time the RA cytotoxicity has been lowered by conjugation to a CPP. It is important, however, to realize that cytotoxicity will vary based on the cargo, the CPP being used, and the site of conjugation [88]. The drastic change in cytotoxicity was thought to be caused by altered bioactivity, which is always a concern when making covalent conjugations to a biomaterial [89]. Furthermore, the RA-PepB_3_ was observed to localize to lysosomal compartments after uptake, which is a major challenge when using CPPs for therapeutic delivery [90]. However, some of the RA-PepB_3_ was found in the cytosol after three days, suggesting endosomal escape (Figure 8). Differentiation results indicate that ReNcell VM differentiated with 0.1 µM RA or RA-PepB_3_ had statistically similar β-III tubulin and MAP2 protein expression levels after four weeks (Figure 11). Due to the decreased toxicity of RA after conjugation to PepB, the possibility of accelerated differentiation was also explored. Using a 5-fold higher concentration (0.5 µM RA-PepB_3_), a 1.5-fold increase in β-III tubulin expression was observed after just one week of differentiation (Figure 12). This level was comparable to using 0.1 µM RA, and decreased bioactivity is a known effect resulting from the conjugation of bioactive molecules to polymers [91]. However, further increasing the concentration to 50-fold (5 µM RA-PepB_3_) resulted in a 2.2-fold increased β-III tubulin expression after just one week. These results suggest that accelerated neuronal differentiation can be achieved with higher concentrations of RA being delivered (when using PepB_3_). It has not been possible to utilize RA (by itself) at high concentrations due to solubility and cytotoxicity issues.

## 5. Conclusions

In summary, a novel cell penetrating peptide called PepB was synthesized. PepB has a repeat sequence consisting of six amino acids (AAAAEK), which was able to internalize at various levels into ReNcell VM human neural stem cells, based on the number of repeats. When retinoic acid was covalently conjugated to PepB_3_, the uptake was significantly increased. After 4 weeks, the cells differentiated with RA-PepB_3_ had comparable expression levels of β-III tubulin and MAP2 to the cells differentiated with RA alone (at low concentrations). Importantly, conjugation to PepB_3_ attenuated the cytotoxicity observed using higher (>2.5 µM) concentrations of RA. Further investigations are underway to see whether increased PepB repeats will continue to result in an increased uptake. Finally, it was possible to use a 50-fold higher concentration of RA-PepB_3_ without cytotoxic effects and with increased levels of neuronal differentiation after just one week. These results suggest that further development can lead to accelerated neuronal differentiation utilizing both PepB series and RA.

## 6. Patents

There is a pending patent resulting from the work reported in this manuscript.

## Figures and Tables

**Table 1:** Molecular weight details for PepB variants. Acetyl, palmitoyl, and retinoyl tail chemical (molecular) structures are provided in Figure 1. Please note that there is an extra alanine at the C-terminus and an extra lysine (for dye conjugation) at the N-terminus. * PepB variant ultimately utilized for the differentiation study detailed in Section 3.5. RA: retinoic acid.

PepB Repeat Structures (*n*)	NH_2_-Terminus	Predicted Mass (Da)	Abbreviated Name
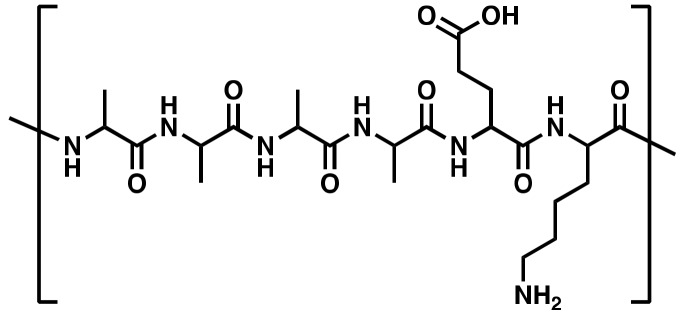	Acetyl	1212.60	Ac-PepB_1_
	Palmitoyl	1409.76	C16-PepB_1_
	Retinoyl	1452.79	RA-PepB_1_
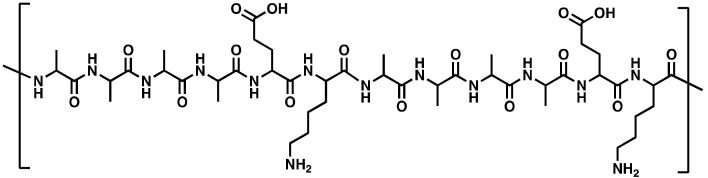	Acetyl	1753.89	Ac-PepB_2_
	Palmitoyl	1950.11	C16-PepB_2_
	Retinoyl	1994.08	RA-PepB_2_
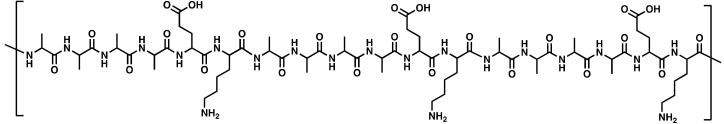	Acetyl	2295.18	Ac-PepB_3_
	Palmitoyl	2491.40	C16-PepB_3_
	Retinoyl	2535.36	RA-PepB_3_*

**Table 2:** Exact mass analysis from mass spectrometry (MS). All PepB variants had matching *m*/*z*. * PepB variant ultimately utilized for the differentiation study detailed in Section 3.5. ESI-ToF: electrospray ionization time-of-flight. MALDI-ToF: matrix-assisted laser deposition/ionization time-of-flight.

Abbreviated Name	PepB Repeats (*n*)	NH_2_-Terminus Tail	Predicted Mass (Da)	ESI-ToF Measured Mass (*m*/*z*)	MALDI-ToF Measured Mass
Ac-PepB_1_	1	Ac	1212.60	606.81	1212.66
C16-PepB_1_	1	C16	1409.76	704.92	1409.73
RA-PepB_1_	1	RA	1452.79	728.92	1457.01
Ac-PepB_2_	2	Ac	1753.89	585.64	1754.53
C16-PepB_2_	2	C16	1950.11	651.04	1950.52
RA-PepB_2_	2	RA	1994.08	667.04	1998.38
Ac-PepB_3_	3	Ac	2295.18	574.80	2295.28
C16-PepB_3_	3	C16	2491.40	623.85, 831.47	2491.94
RA-PepB_3_*	3	RA	2535.36	635.60	2539.78
